# Bayesian phylodynamic inference with complex models

**DOI:** 10.1371/journal.pcbi.1006546

**Published:** 2018-11-13

**Authors:** Erik M. Volz, Igor Siveroni

**Affiliations:** Department of Infectious Disease Epidemiology and the MRC Centre for Global Infectious Disease Analysis, Imperial College London, London, United Kingdom; University of Technology Sydney, AUSTRALIA

## Abstract

Population genetic modeling can enhance Bayesian phylogenetic inference by providing a realistic prior on the distribution of branch lengths and times of common ancestry. The parameters of a population genetic model may also have intrinsic importance, and simultaneous estimation of a phylogeny and model parameters has enabled phylodynamic inference of population growth rates, reproduction numbers, and effective population size through time. Phylodynamic inference based on pathogen genetic sequence data has emerged as useful supplement to epidemic surveillance, however commonly-used mechanistic models that are typically fitted to non-genetic surveillance data are rarely fitted to pathogen genetic data due to a dearth of software tools, and the theory required to conduct such inference has been developed only recently. We present a framework for coalescent-based phylogenetic and phylodynamic inference which enables highly-flexible modeling of demographic and epidemiological processes. This approach builds upon previous structured coalescent approaches and includes enhancements for computational speed, accuracy, and stability. A flexible markup language is described for translating parametric demographic or epidemiological models into a structured coalescent model enabling simultaneous estimation of demographic or epidemiological parameters and time-scaled phylogenies. We demonstrate the utility of these approaches by fitting compartmental epidemiological models to Ebola virus and Influenza A virus sequence data, demonstrating how important features of these epidemics, such as the reproduction number and epidemic curves, can be gleaned from genetic data. These approaches are provided as an open-source package *PhyDyn* for the BEAST2 phylogenetics platform.

This is a *PLOS Computational Biology* Software paper.

## Introduction

Mechanistic models guided by expert knowledge can form an efficient prior on epidemic history when conducting phylodynamic inference with genetic data [1]. Parameters estimated by fitting mechanistic models, such as the reproduction number *R*_0_, are important for epidemic surveillance and forecasting. Compartmental models defined in terms of ordinary or stochastic differential equations are the most common type of mathematical infectious disease model, but in the area of phylodynamic inference, non-parametric approaches such as skyline coalescent models [2] or sampling-birth-death models [3] are more commonly used. Methods to translate compartmental infectious disease models into a population genetic framework have been developed only recently [4–8]. We address the gap in software tools for epidemic modeling and phylogenetic inference by developing a BEAST2 package, *PhyDyn*, which includes a highly-flexible markup language for defining compartmental infectious disease models in terms of ordinary differential equations. This flexible framework enables phylodynamic inference with the majority of published compartmental models, such as the common susceptible-infected-removed (SIR) model [9] and its variants, which are often fitted to non-genetic surveillance data. The *PhyDyn* model definition framework supports common mathematical functions, conditional logic, vectorized parameters and the definition of complex functions of time and/or state of the system. The *PhyDyn* package can make use of categorical metadata associated with each sampled sequences, such as location of sampling, demographic attributes of an infected patient (age, sex), or clinical biomarkers. Phylogeographic models designed to estimate migration rates between spatial demes [10–12] are special cases within this modeling framework, and more complex phylogeographic models (e.g. time-varying or state-dependent population size or migration rates) can also be easily defined in this framework.

The development of *PhyDyn* was influenced by and builds upon previous efforts to incorporate mechanistic infectious disease models in BEAST2. The *bdsir* BEAST2 package [13] implements a simple SIR model which is fitted using an approximation to the sampling-birth-death process. The *phylodynamics* BEAST2 package [14] includes simple deterministic and stochastic SIR models which can be fitted using coalescent processes. More recently, the *EpiInf* package has been developed which can fit stochastic SIR models using an exact likelihood with particle filtering [15]. These epidemic modeling packages are, however, limited to unstructured populations (no spatial, risk-group, or demographic population heterogeneity). Other packages have been developed for spatially structured populations with a focus on phylogeographic inference, especially with the aim of estimating pathogen migration rates between discrete spatial locations [16]. The *MultiTypeTree* BEAST2 package [10] implements the exact structured coalescent model with multiple demes and with constant effective population size in each deme and constant migration rates between demes. Two BEAST2 packages, *BASTA* [17] and *MASCOT* [11] have been independently developed to use fast approximate structured coalescent models. These packages mirror the functionality of *MultiTypeTree* but include approximations to reduce computational requirements, enabling estimation of time-invariant effective population sizes and migration rates between spatial demes.

The *PhyDyn* BEAST2 package provides new functionality to the BEAST2 phylogenetics platform by implementing a much more complex family of structured coalescent models. In a general compartmental model, neither the effective population size nor migration rate between demes need be constant, and in more general frameworks, coalescence is also allowed between lineages occupying different demes. The package includes a flexible markup language for defining compartmental models within the BEAST2 XML. This includes common mathematical functions making it simple to develop models which incorporate seasonality or which deviate from the simplistic mass-action premise of basic SIR models. Models defined with this special syntax can be directly incorporated into BEAST2 XML files for easily reproducing and modifying analyses. The *PhyDyn* model markup language supports vectorised parameters (e.g. an array of transmission rates or population sizes) and simple conditional logic statements, so that epidemic dynamics can change in a discrete fashion, such as from year to year or in response to a public-health intervention. Commonly used phylogeographic models based on the structured coalescent are a special case of the general compartmental models implemented in the *PhyDyn* package, and extensions to the basic phylogeographic model can be implemented, such as by allowing effective population size to vary through time in each deme according to a mechanistic model.

## Design and implementation

In this framework, first described in [5], we define deterministic demographic or epidemiological processes of a general form which includes the majority of compartmental models used in mathematical epidemiology and ecology. Defining compartmental models within this form facilitates interpretation of the population genetic model developed in the next section. Let there be *m* demes, and the population size within each deme is given by the vector-valued function of time *Y*_1:*m*_(*t*). We may also have *m*′ dynamic variables $Y_{1:m^{\prime }}^{\prime }\left(t\right)$ which are not demes (hence do not correspond to the state of a lineage), but which may influence the dynamics of *Y*. The dynamics of *Y* arise from a combination of *births* between and within demes, *migrations* between demes, and *deaths* within demes. We denote these as deterministic matrix-valued functions of time and the state of the system, following the framework in [5]:

- Births: *F*_1:*m*,1:*m*_(*t*, *Y*, *Y*′). This may also correspond to transmission rates between different types of hosts in epidemiological models.
- Migrations: *G*_1:*m*,1:*m*_(*t*, *Y*, *Y*′). These rates may have non-geographic interpretations in some models (e.g. aging, disease progression).
- Deaths: *μ*_1:*m*_(*t*, *Y*, *Y*′). These terms may also correspond to recovery in epidemiological models.

The elements *F*_*kl*_(⋯) describe the rate that new individuals in deme *l* are generated by individuals in deme *k*. For example, this may represent the rate that infected hosts of type *k* transmit to susceptible hosts of type *l*. The elements *G*_*kl*_(⋯) represent the rate that individuals in deme *k* change state to type *l*, but these rates do not describe the generation of new individuals. With the above functions defined, the dynamics of *Y*(*t*) can be computed by solving a system of *m* + *m*′ ordinary differential equations:

$${\dot{Y}}_{k}\left(t\right)=-\mu_{k}\left(t\right)+\sum_{l=1}^{m}\left(F_{lk}\left(t\right)+G_{lk}\left(t\right)-G_{kl}\left(t\right)\right)$$ (1)

The *PhyDyn* package model markup language requires specifying the non-zero elements of *F*(*t*), *G*(*t*) and *μ*(*t*). There are multiple published examples of simple compartmental models developed in this framework [18–23]. In the following sections, we give examples of simple compartmental models related to infectious diease dynamics and show how these models can be defined within this framework and code samples are also provided online. We provide examples of models fitted to data from seasonal human Influenza virus and Ebola virus as well as a simulation study.

### Seasonal human influenza model

**Fig 1:**
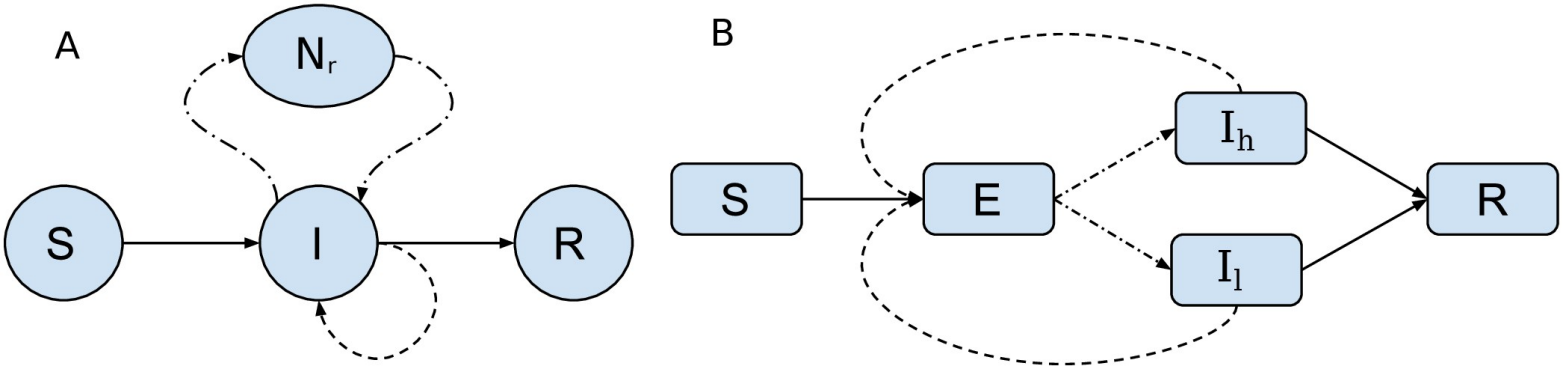
Compartmental diagram representing structure of models for seasonal human Influenza (A) and Ebola virus model (B). Solid lines represent flux of hosts between different categories. Dash lines represent migration. Dotted lines represent births (transmission).

We model a single season of Influenza A virus (IAV) H3N2 and apply this model to 102 HA-1 sequences collected between 2004 and 2005 in New York state [24, 25]. We build on a simple susceptible-infected-recovered (SIR) model which accounts for importations of lineages from the global reservoir of IAV, which we will see is a requirement for good model fit to these data (Fig 1). This model has two demes: The first deme corresponds to IAV lineages circulating in New York, and the second deme corresponds to the global IAV reservoir. The global reservoir will be modeled as a constant-size coalescent process. Within New York state, new infections are generated at the rate *βI*(*t*)*S*(*t*)/*N* where *β* is the per-capita transmission rate per day, *I*(*t*) is the number of infected and infectious hosts, *S*(*t*) is the number of hosts susceptible to infection, and *N* = *S* + *I* + *R* is the population size. *R*(*t*) denotes the number of hosts that have been infected and are now immune to this particular seasonal variant. With the above definitions, we define the matrix-valued function of time:

$$\begin{array}{cc} F(t) & =[\begin{array}{cc} \beta I(t)S(t)/N(t) & 0\\ 0 & \gamma N_{r} \end{array}]. \end{array}$$ (2)

Note that births within the reservoir do not vary through time and depend on the effective population size in that deme *N*_*r*_.

Additionally, we model *deaths* from the pool of infected using

$$\begin{array}{cc} \mu (t) & =[\begin{array}{c} \gamma I(t)\\ \gamma N_{r} \end{array}]. \end{array}$$ (3)

Births balance deaths in the reservoir population.

Finally, we model a symmetric migration process between the reservoir and New York:

$$\begin{array}{c} G\left(t\right)=[\begin{array}{cc} 0 & \eta I(t)\\ \eta I(t) & 0 \end{array}], \end{array}$$ (4)

where *η* is the per-capita migration rate. Note that migration between the reservoir and New York are balanced and do not effect the dynamics of *I*(*t*) over time.

*PhyDyn* code for defining these equations can be found at https://github.com/mrc-ide/PhyDyn/wiki/Influenza-Example.

These three processes lead to the following differential equation for the dynamics of *I*(*t*):

$$\begin{array}{c} \dot{I}\left(t\right)=\beta I\left(t\right)S\left(t\right)/N\left(t\right)-\gamma I\left(t\right). \end{array}$$

Below, we show a fit of this model where the following parameters are estimated:

- Migration rate *η*; prior (events per year): lognormal (log mean = 1.38, log sd = 1)
- Recovery rate *γ*; prior (events per year): lognormal(log mean = 4.8, log sd = 0.25)
- Reproduction number *R*_0_ = *β*/*γ*; prior: lognormal(log mean 0, log sd = 1)
- Reservoir size *N*_*r*_; prior: lognormal(log mean = 9.2, log sd = 1)
- Initial number infected in September 2004; prior: lognormal(log mean = 0, log sd = 1)
- Initial number susceptible in September 2004; lognormal(log mean = 9.2, log sd = 1)

Note that the model only had one informative prior, which was for the recovery rate, and was based on the previous study of viral shedding by Cori et al. [26] Previous work [27] on identifiability of parameters in phylodynamic models has shown that it is generally impossible to simultaneously infer transmssion and recovery rates without additional data or strong assumptions about the sampling rate.

### Ebola virus in Western Africa

We develop a susceptible-exposed-infected-recovered (SEIR) model (Fig 1) for the 2014-2015 Ebola Virus (EBOV) epidemic in Western Africa and apply this model to phylogenies previously estimated by Dudas et al. [28]. Phylogenies estimated by Dudas are randomly downsampled to *n* = 400 to alleviate computational requirements.

According to the SEIR model, infected hosts progress from an uninfectious exposed state (E) to an infectious state (I) at rate *γ*_0_ which influences the generation-time distribution of the epidemic. Infectious hosts die or recover at the rate *γ*_1_. The SEIR model has the following form:

$$\begin{array}{c} \frac{d}{dt}E=\beta \left(t\right)I\left(t\right)-\gamma_{0}E\left(t\right),\\ \frac{d}{dt}I=\gamma_{0}E\left(t\right)-\gamma_{1}I\left(t\right). \end{array}$$ (5)

where *β*(*t*) is the per-capita transmission rate per year. In a typical mass-action model, we would have *β*(*t*) ∝ *S*(*t*)/(*S*(*t*) + *E*(*t*) + *I*(*t*) + *R*(*t*)), however in order to demonstrate the flexibility of this modeling framework, we will instead use a simple linear function, *β*(*t*) = *at* + *b*, and in general a wide variety of parametric and non-parametric functions could be used within the BEAST2 package to model the force of infection. In addition to demonstrating the flexibility of *PhyDyn*, we chose the affine transmission rate model because the mass action assumption is unrealistic and unnecessary. The number of susceptible individuals was never a limiting factor in this epidemic and incidence declined primarily in response to public health interventions.

There are two demes in this model corresponding to the potential states of an infected hosts. The birth matrix with demes in the order (*E*, *I*) is

$$\begin{array}{cc} F(t) & =[\begin{array}{cc} 0 & 0\\ \beta (t)I(t) & 0 \end{array}]. \end{array}$$ (6)

The migration matrix encapsulates all processes which may change the state of a lineage without leading to coalescence of lineages, and this includes progression from E to I:

$$\begin{array}{cc} G(t) & =[\begin{array}{cc} 0 & \gamma_{0}E\left(t\right)\\ 0 & 0 \end{array}] \end{array}$$ (7)

And finally removals are modeled using

$$\begin{array}{cc} \mu (t) & =[\begin{array}{c} 0\\ \gamma_{1}I\left(t\right) \end{array}]. \end{array}$$ (8)

Note that the parametric description of *β*(*t*) does not require us to model dynamics of *S*(*t*) or *R*(*t*).

*PhyDyn* code for defining these equations can be found at https://github.com/mrc-ide/PhyDyn/wiki/Ebola-Example.

The parameters estimated and priors for this model are

- *β*(*t*) slope *a*, prior: Normal(0, 40)
- *β*(*t*) intercept *b*, prior: lognormal(log mean = 4.6, log sd = 1)
- Initial number infected (beginning of 2014), prior: lognormal (log mean = 0, log sd = 1)

In order to reconstruct an epidemic trajectory which closely matched the absolute numbers of cases through time, we include additional variables that could influence the relationship between effective population size and the true number of infected hosts. For this purpose we developed a second EBOV model which included higher variance in the offspring distribution, reasoning that a higher variance in the number of transmissions per infected case would lead to higher estimates of the epidemic size [29]. The superspreading model (Fig 1) includes two infectious compartments, *I*_*l*_ and *I*_*h*_, with per-capita transmission rates *β*(*t*) and *τβ*(*t*) respectively. The factor of *τ* > 1 represents a transmission risk ratio for the second infectious deme. We specify that a constant fraction *p*_*hr*_ progress from *E* to *I*_*h*_, with the remainder going to *I*_*l*_. With demes in the order (*E*, *I*_*l*_, *I*_*h*_), the birth, migration, and death matrices for the superspreading model are as follows:

$$\begin{array}{cc} F(t) & =[\begin{array}{ccc} 0 & 0 & 0\\ \beta \left(t\right)I_{l}\left(t\right) & 0 & 0\\ \tau \beta \left(t\right)I_{h}\left(t\right) & 0 & 0 \end{array}], \end{array}$$ (9)

$$\begin{array}{cc} G(t) & =[\begin{array}{ccc} 0 & \left(1-p_{hr}\right)\gamma_{0}E\left(t\right) & p_{hr}\gamma_{0}E\left(t\right)\\ 0 & 0 & 0\\ 0 & 0 & 0 \end{array}], \end{array}$$ (10)

$$\begin{array}{cc} \mu (t) & =[\begin{array}{c} 0\\ \gamma_{1}I_{l}\left(t\right)\\ \gamma_{1}I_{h}\left(t\right) \end{array}]. \end{array}$$ (11)

Additional parameters and priors for the superspreading model are

- *τ*, prior: lognormal(log mean = 1, log sd = 1)
- *p*_*hr*_, fixed at 20%

Note that we used an uninformative prior for *τ* as our previous studies with a related model showed that superspreading parameters are potentially identifiable [21]. This model did not include geographic structure, although the samples were geographically diverse, and some model-misspecification bias is anticipated if migration between spatial demes is sufficiently small.

### Simulation model

We developed a simulation model with four demes in order to evaluate the ability of BEAST2 to identify and estimate birth rates, migration rates, and transmission risk ratios. This model includes two types of hosts, with low and high transmission risk. Additionally, each type of host progresses through two stages of infection, where the first stage is short but has higher transmission rate. The four demes are denoted *Y*_0*l*_, *Y*_1*l*_, *Y*_0*h*_, *Y*_1*h*_ where the first subscript denotes stage of infection and the second subscript denotes transmission risk level. The model is illustrated as S1 Fig.

The birth matrix is:

$$\begin{array}{cc} F(t) & =[\begin{array}{cccc} p_{l}f\left(t\right)w_{0}Y_{0l}\left(t\right)/W\left(t\right) & 0 & \left(1-p_{l}\right)f\left(t\right)w_{0}Y_{0l}\left(t\right)/W\left(t\right) & 0\\ p_{l}f\left(t\right)Y_{1l}\left(t\right)/W\left(t\right) & 0 & \left(1-p_{l}\right)f\left(t\right)Y_{1l}\left(t\right)/W\left(t\right) & 0\\ p_{l}f\left(t\right)w_{0}w_{h}Y_{0h}\left(t\right)/W\left(t\right) & 0 & \left(1-p_{l}\right)f\left(t\right)w_{0}w_{h}Y_{0l}\left(t\right)/W\left(t\right) & 0\\ p_{l}f\left(t\right)w_{h}Y_{1h}\left(t\right)/W\left(t\right) & 0 & \left(1-p_{l}\right)f\left(t\right)w_{h}Y_{1h}\left(t\right)/W\left(t\right) & 0 \end{array}]. \end{array}$$ (12)

In this model, a proportion *p*_*l*_ of all transmissions go to the low risk group. Transmissions from stage 1 are proportional to the transmission risk ratio *w*_0_ > 1. Transmissions from the high risk group are proportional to the transmission risk ratio *w*_*h*_ > 1. The variable *W*(*t*) = *w*_0_*Y*_0*l*_ + *Y*_1*l*_ + *w*_0_*w*_*h*_*Y*_0*h*_ + *w*_*h*_*Y*_1*h*_ normalizes the proportion of transmissions attributable to each deme. The variable *f*(*t*) gives the total number of transmissions per unit time, and for this we use a SIRS model:

$$\begin{array}{c} f\left(t\right)=\beta \left(Y_{0l}+Y_{1l}+Y_{0h}+Y_{1h}\right)S/N, \end{array}$$

where *S*(*t*) is the number susceptible governed by:

$$\begin{array}{c} \dot{S}=-f\left(t\right)+\eta S\left(0\right)-\eta S\left(t\right), \end{array}$$

and, *η* is the per-capita rate of non-disease related mortality.

The migration matrix captures the disease stage-progression process:

$$\begin{array}{cc} G(t) & =[\begin{array}{cccc} 0 & \gamma_{0}Y_{0l}\left(t\right) & 0 & 0\\ 0 & 0 & 0 & 0\\ 0 & 0 & 0 & \gamma_{0}Y_{0h}\left(t\right)\\ 0 & 0 & 0 & 0 \end{array}]. \end{array}$$

The death matrix is

$$\begin{array}{cc} \mu (t) & =[\begin{array}{c} \eta Y_{0l}\left(t\right)\\ \left(\eta +\gamma_{1}\right)Y_{1l}\left(t\right)\\ \eta Y_{0h}\\ \left(\eta +\gamma_{1}\right)Y_{1h}\left(t\right) \end{array}]. \end{array}$$

*PhyDyn* code for implementing this model can be found at https://git.io/ftjg5.

To generate simulated data, we simulated epidemics using Gillespie’s exact algorithm over a discrete population and an initial susceptible population of two or five thousand individuals. A random sample of *n* = 250 or 500 was collected between times 95 and 250 and the history of transmissions was used to reconstruct a genealogy. *PhyDyn* was then used to estimate

- *β*, prior: lognormal (log mean = -1.6, log sd = 0.5)
- *w*_0_, prior: uniform(0, 50)
- *w*_*h*_, prior: uniform(0, 50)
- The initial number infected, prior: lognormal (log mean = 0, log sd = 1)

Note that *PhyDyn* is fitting deterministic models to data generated from a noisy stochastic process and some error should be expected due to this approximation. S2 Fig shows a comparison of a single noisy simulated trajectory and a solution of the deterministic model under the true parameters. All simulation code and BEAST2 XML files are available at https://github.com/emvolz/PhyDyn-simulations.

### Modeling the coalescent process conditioning on a complex demographic history

The coalescent likelihood is based on the conditional density of a genealogy given epidemic and demographic parameters. In BEAST2, the coalescent likelihood is used in tandem with evolutionary models that provide the probability density of a genealogy given a genetic sequence alignment and evolutionary parameters. But the coalescent likelihood can also be used if a time-scaled phylogeny has been estimated independently.

Various approximations have been developed for computing the density of a genealogy conditional on a complex demographic history. These differ by the extent to which they account for correlation between co-existing lineages in the genealogy, the extent to which they account for finite size of the population, and the extent to which they account for differences in coalescent rates in different demes. There is a speed/bias tradeoff between these approximations, and consequently *PhyDyn* makes several model variations available. The choice of likelihood approximation depends on time and computational resources available, sample size, and model complexity. Three likelihood approximations are described in S1 Text, and we derive a new approximation which has shown greater accuracy in some situations.

The structured coalescent model in [5] which inspired the development of *PhyDyn* did not account for all correlations between co-existing lineages or all effects stemming from disparate coalescent rates between demes. In [20], a fast likelihood approximation was derived which better accounted for potential bias resulting from highly-disparate coalescent rates in different demes. This model, denoted *QL*, also makes strong approximations regarding lineage independence: In every internode interval, all lineages are updated according to a linear transformation which varies through time but not between lineages. These issues were investigated as a source of bias in the context of phylogeographic models in [30], where yet another likelihood approximation was proposed for models with constant population size and constant migration rates.

In the PhyDyn package, we have developed likelihood approximations based on *QL* which better account for correlation between lineages. These models, denoted *PL1* and *PL2*, work by solving a system of differential equations for each lineage while including terms similar to those in the *QL* model that account for disparate coalescent rates between demes. While these models are demonstrably more accurate in simulation studies, they require more computation. All three likelihood approximations are provided in the PhyDyn package. The new *PL2* model is the suggested default model choice, however the *QL* model may be preferred for some large datasets or when fitting complex models due to computational advantages. The new models are derived in S1 Text.

## Results

### Human influenza A/H3N2

**Fig 2:**
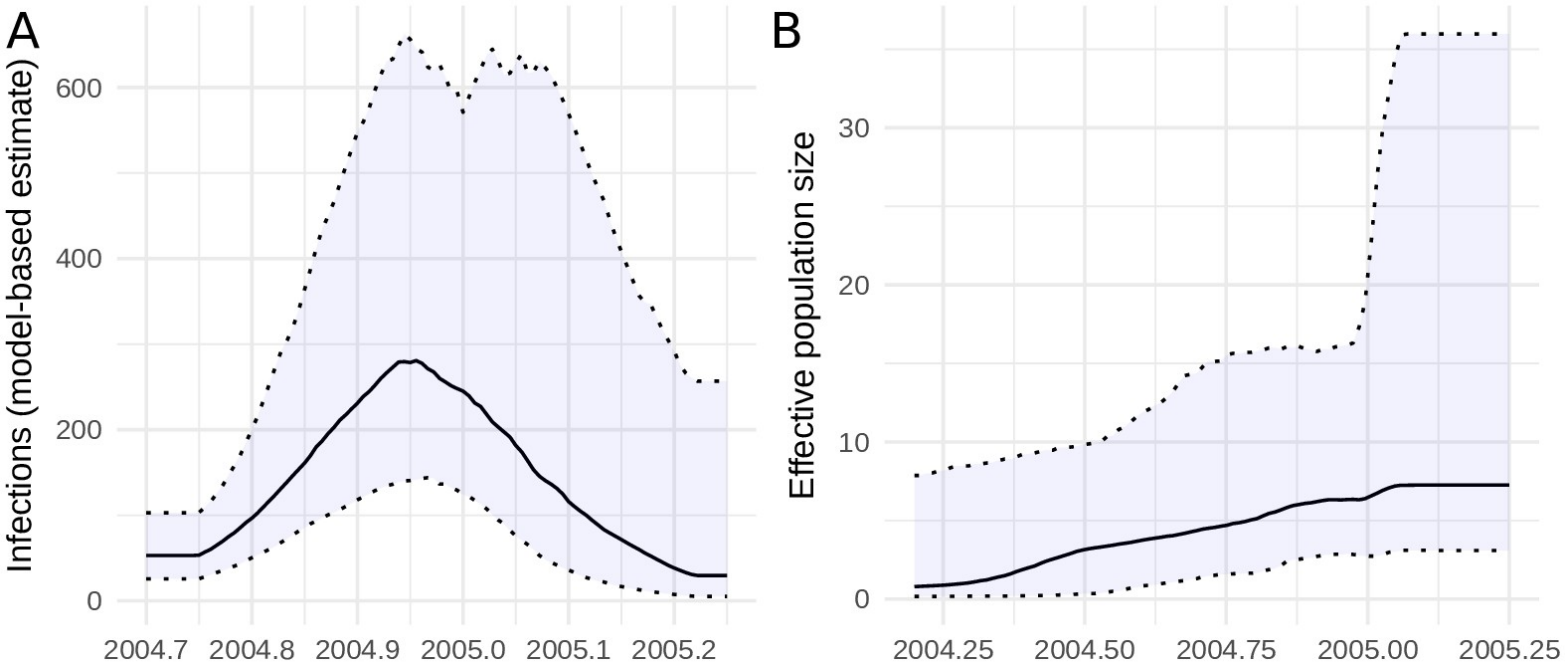
The estimated effective number of H3N2 human influenza infections in 2004-2005 in New York State. A. Estimates obtained using the parametric seasonal influenza model described in the text. B. Effective population size estimated using a conventional Bayesian skyline analysis.

The seasonal influenza SIR model which accounts for importations from the global reservoir was applied to 102 HA/H3N2 sequences collected from New York state during the 2004-2005 flu season. These data were previously analyzed using non-parametric models by [24]. Fig 2 shows the estimated posterior effective number of infections over the course of the influenza season, and the time of peak prevalence is correctly identified around the end of 2004. We also compared the model-based estimates to estimates generated in BEAST2 using a conventional non-parametric Bayesian skyline model which is also shown in Fig 2. The skyline model does not detect a decrease in prevalence towards the end of the influenza season and does not identify the time of peak prevalence. We carried out a further comparison with estimates using a GMRF skyride model fitted in BEAST 1.8 [31, 32] (S3 Fig). The skyride model correctly detected a peak in *N*_*e*_ in late 2014 and subsequent decline, however variation *N*_*e*_(*t*) was quite small relative to uncertainty in the credible intervals. The peak of *N*_*e*_ was slightly too early, and *N*_*e*_ was also larger prior to the 2014-15 influenza season due to the effects of unmodeled lineage importation from outside New York. Skyline and skyride analysis data and files are available at https://github.com/emvolz/nyflu-skyline. Use of a well-specified parametric compartmental model imposes a strong prior on the epidemic trajectory which leads to the correct identification of the shape and timing of the epidemic curve.

We estimated the reproduction number *R*_0_ = 1.16 (95%CI: 1.07-1.30). This value is similar to many previous estimates based on non-genetic data for seasonal influenza in humans which according to the recent review in [33] have an interquartile range of 1.18-1.27 for H3N2. Bettancourt et al. [34] estimated *R*_0_ = 1.22 for the 2004-05 H3N2 seasonal influenza epidemic in the entire USA using weekly case report data. An uninformative prior was used for *R*_0_ in the *PhyDyn* analysis.

### Ebola virus in Western Africa

We applied the SEIR and superspreading-SEIR models to Ebola virus phylogenies based on data first described by [28] and subsequently analyzed in [35]. These phylogenies were estimated from whole genome sequences collected 2014-2015 during the West African Ebola epidemic. We derived the maximum clade credibility tree from the analysis by [28] and extracted a subtree based on sampling four hundred lineages at random. The *PhyDyn* package was used to fit the models with fixed tree topologies and branch lengths. Co-estimating the phylogeny and epidemic parameters is possible and may lead to more robust credible intervals because the tree prior can influence the topology of the estimated posterior distribution of trees, but this would also require substantialy more computational effort. The trees were fixed in this analyis in order to facilitate comparisons with other software and because of computational tradeoffs. With this fixed tree, *PhyDyn* executes approximately one million MCMC steps per 17 hours using a typical CPU. We also ran the analysis using a fixed tree estimated by maximum likelihood and the *treedater* R package as described in [35], finding similar results.

**Fig 3:**
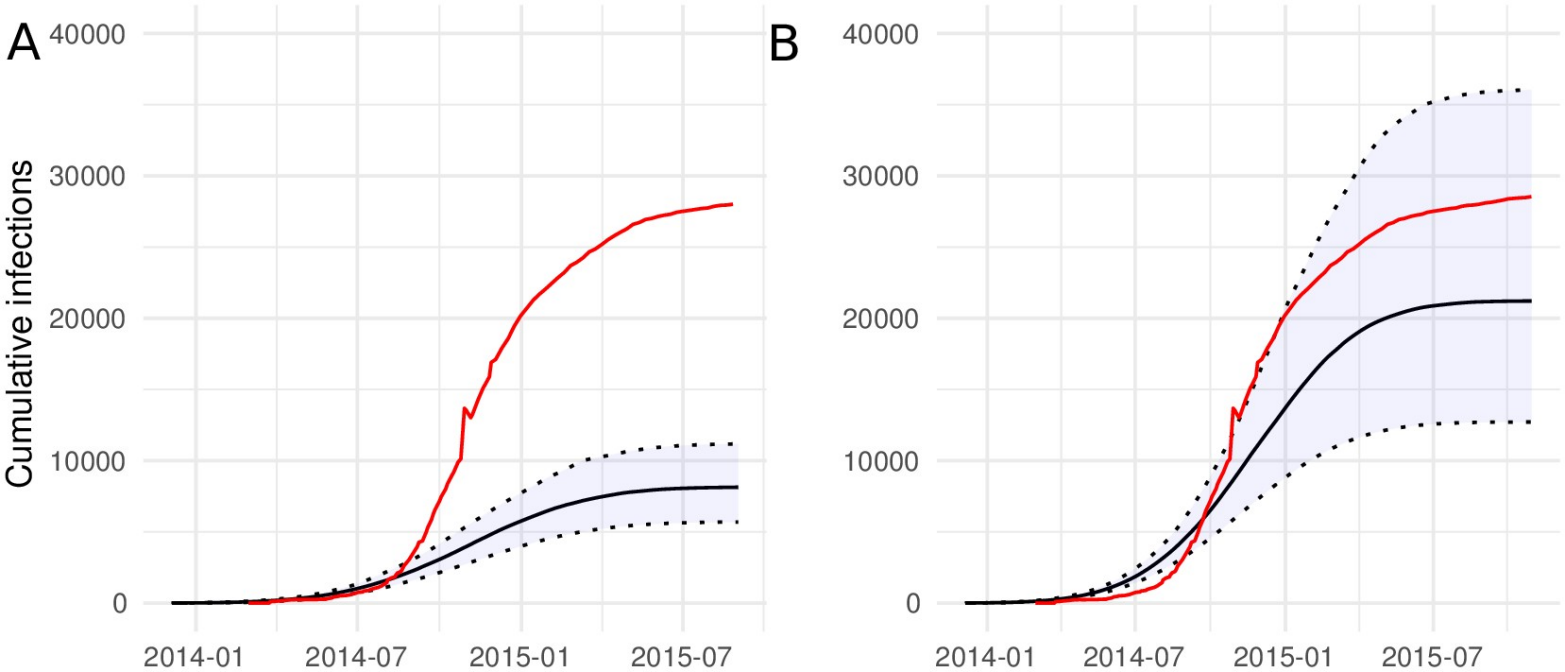
Model-based estimates of cumulative infections through time for the 2014-15 Ebola epidemic in Western Africa. Estimates are shown for the SEIR model (A) and the model which includes super-spreading (B). The red line show the cumulative number of cases reported by WHO [35].

**Fig 4:**
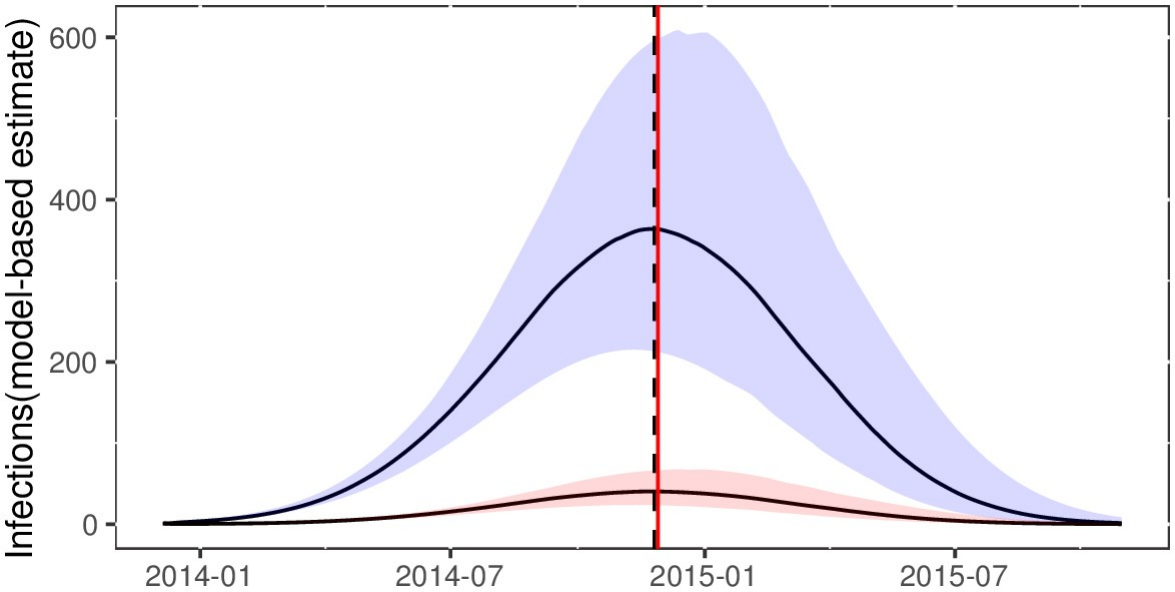
Estimated effective number of infections through time using the superspreading SEIR model for the 2014-15 Ebola epidemic in Western Africa. The red vertical line shows the time of peak prevalence inferred from WHO case reports. The vertical dashed line shows the model estimated time of peak prevalence. The red trajectory shows the proportion of infections in the high-transmission-rate compartment.

The transmission rate (per year) *β*(*t*) was estimated as a linear function with slope -13.22(95%CI:-14.4587- -12.036) and intercept 85.1(95% CI: 83.93-86.16). We estimated similar reproduction numbers using both models. With the SEIR model, we compute *R*_0_ = *β*(*t*)/*γ*_1_. We estimate *R*_0_ = 1.47(95%CI: 1.41-1.53). With the superspreading-SEIR model, we have a similar estimate of *R*_0_ = 1.52(95%CI:1.48-1.54). Note that uninformative priors were used for parameters determining *R*_0_. As anticipated, the model fits provide substantially different estimates of the cumulative number of infections. Fig 3 shows the estimated cumulative infections through time using both models alongside the cumulative number of cases reported by WHO and compiled by the US CDC [35]. Both models provide similar estimates regarding the relative numbers infected through time and the time of epidemic peak. Using the superspreading model, the time of peak incidence is estimated to have occurred on November 25, 2014. According to WHO reports, this occurred only three days later on November 28 (Fig 4.

Estimates of cumulative infections with the superspreading model are consistent with WHO data, whereas results with the SEIR model are not. The superspreading model accomodates an over-dispersed offspring distribution (the number of transmission per infection), thereby decreasing effective population size per number infected and yielding larger estimates for the number infected [29]. We estimate the transmission risk ratio parameter (ratio of transmission rates between high and low compartments) to be 8.1 (95%CI: 6.68-10.73). This implies that a minority of 10% of infected individuals are responsible for 43%-54% of infections.

### Simulations

**Fig 5:**
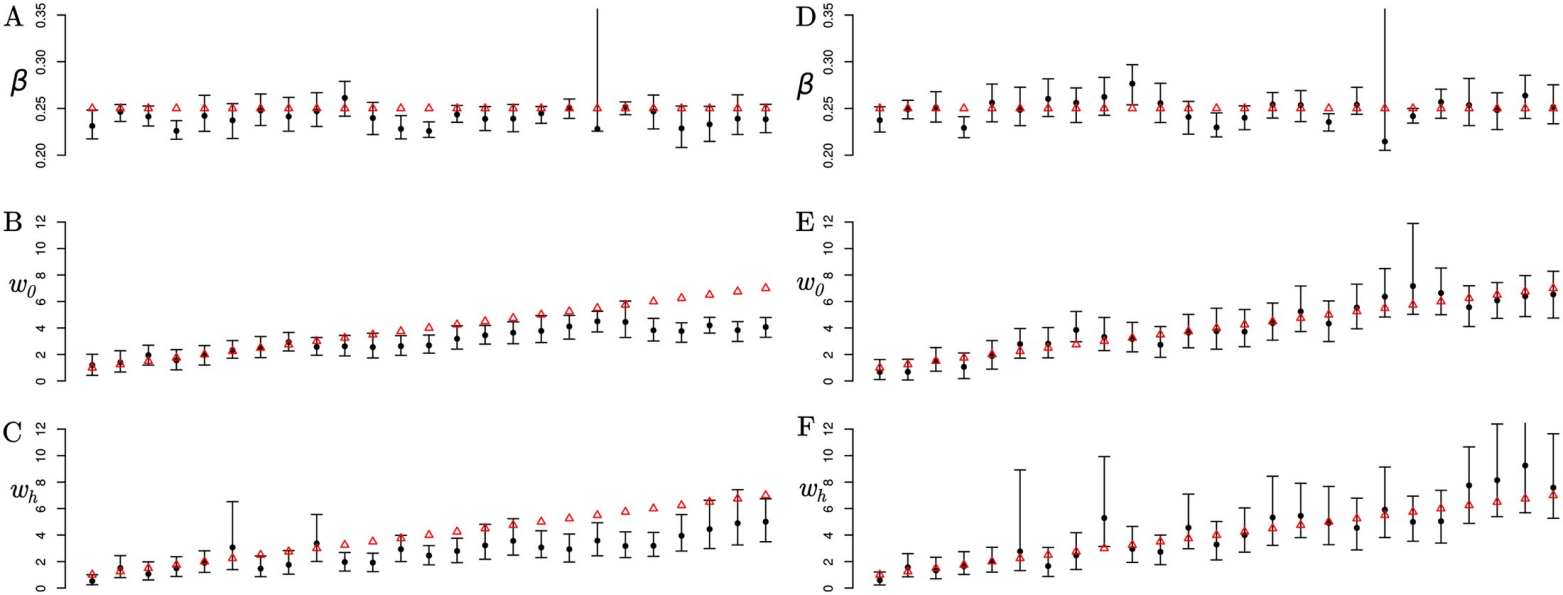
Parameter estimates and credible intervals for 25 simulations with variable transmission risk ratos. The red points show true parameter value. The parameter *β* is the per-capita transmission rate, and *w*_0_ and *w*_*h*_ are respectively the transmission risk ratios in the first stage of infection and the high risk group (cf. Eq 12). A-C: Results generated using the *QL* model. D-F: Results generated using the *PL2* model. There is one outlier simulation where the transmission rate parameter could not be estimated precisely and upper bound of the CI was > 70% using both methods.

With simulated tree data, *PhyDyn* recovers the correct transmission risk ratios and transmission rates, although performance depends on which structured coalescent model is used. Fig 5 compares estimates across 25 simulations using *PL2* and *QL* models on epidemics with 5,000 initial susceptible individuals and a sample size of 500 sampled heterochronously shortly after epidemic peak. The transmission risk ratio parameters were varied across simulations between and the per-capita transmission rate was kept constant. S4 Fig shows performance of the *PL1* model which was similar to *PL2* but had slightly higher bias and lower posterior coverage of true parameters. Results for a smaller and noisier epidemic (2000 initial susceptibles) is shown in S5 Fig. The running time of the *QL* model was approximately five times faster than *PL2* which required approximately 12 hours to complete 35,000 MCMC iterations, however *QL* has considerable bias at the upper range of transmission risk ratio parameters and corresponding lower posterior coverage.

Good coverage of parameter estimates with estimated 95% credible intervals was observed with the *PL2* model. Across 75 parameter estimates (three parameters not counting initial conditions and 25 simulations), estimates did not cover the true value 4 times. Bias of the mean posterior estimate was quite small; the largest bias was 0.228 for the *w*_*h*_ parameter which varied across simulations between 1 and 9. In contrast, the *QL* model failed to cover much more frequently, however errors were largely confined to larger risk ratios and *QL* had a tendency to underestimate risk ratios. Greater bias was observed with the *QL* model, with the greatest bias observed for the *w*_*h*_ parameter (mean bias:-0.48). However the *QL* model also had good precision with smaller risk ratios as evidenced in the simulation with smaller population size (S5 Fig). In that case, the *PL2* model showed slight bias towards overestimating risk ratios which may be due to the deterministic approximation to the noisy epidemic. A similar but less pronounced pattern of bias and precision was observed for other parameters. A complete summary of simulation results is available at https://github.com/emvolz/PhyDyn-simulations.

## Availability and future directions

The *PhyDyn* package, source code, documentation and examples can be found at https://github.com/mrc-ide/PhyDyn. The *PhyDyn* package greatly expands the range of epidemiological, ecological, and phylogeographic models that can be fitted within the BEAST2 Bayesian phylogenetics framework. Extensions enabled by this package include models with parametric seasonal forcing, non-constant parametric migration or coalescent rates between demes, state-dependent migration or coalescent rates, and discrete changes in migration or coalescent rates in response to perturbation of the system (e.g. a public health intervention). The package also provides a means of utilizing non-geographic categorical metadata which is usually not considered in phylodynamic analyses, such as clinical or demographic attributes of patients in a viral phylodynamics application [19].

We have demonstrated the utility of this framework using data from Influenza and Ebola virus epidemics in humans, finding epidemic parameters and epidemic trajectories consistent with other surveillance data. In both of these examples, simple structured models were fitted, but notably without using any categorical metadata associated with sampled sequences. This demonstrates potential advantages of structured coalescent modeling even in the absence of informative metadata. In the case of human Influenza A virus, the fitted model included a deme which accounted for evolution in the unsampled global influenza reservoir, which allowed estimation of epidemic parameters within the smaller sub-region which was intensively sampled. The use of a parametric mass-action model allowed *PhyDyn* to correctly detect the time of epidemic peak and epidemic decline, whereas non-parametric skyline methods did not detect epidemic decline in this case. And in the application to the Ebola virus epidemic in Western Africa, models included un-sampled ‘exposed’ categories which accounted for realistic progression of disease among patients, as well as a ‘super-spreading’ compartment which accounted for over-dispersion in the number of transmissions per infected case.

In developing *PhyDyn*, the focus has been on developing a highly flexible framework which is also computationally tractable for moderate sample sizes and model complexity. But flexibility and computational efficiency has come at the cost of some realism, notably in the deterministic nature of the models included in this framework. Future extensions may utilize stochastic epidemic models such as those described by [36]. Other directions for future development include semi-parametric modeling, such as models with a spline-valued force of infection [22] or models utilizing Gaussian processes [37], and approaches for utilizing continuous-valued metadata [38].

## Supporting information

S1 Text Structured coalescent likelihood and approximations. (PDF)

S1 Fig Diagram representing dynamics of simulation model with four demes. This model has two levels of transmission rate (l and h) and two stages of infection with higher transmission in the first stage. Solid lines represents death or stage progression. Dash lines represent transmissions.
(TIF)

S2 Fig Comparison of stochastic and deterministic trajectories. The stochastic epidemic simulation is shown in black and the deterministic ODE model is shown in red.
(TIF)

S3 Fig Effective population size of influenza H3N2 in New York 2014-15 estimated using GMRF skyride. The median posterior estimate is shown in the panel on the left, and the panel on the right shows both the median and 95% credible intervals.
(TIF)

S4 Fig Parameter estimates using the *PL1* coalescent model and credible intervals for 25 simulations with variable transmission risk ratos. The red points show true parameter value. Top: Transmission rate. Middle: Acute stage transmission risk ratio. Bottom: High risk group transmission risk ratio.
(TIF)

S5 Fig Parameter estimates and credible intervals for 20 simulations. The red line shows the true value. A-C: Results generated using the *PL1* model. D-F: Results generated using the *QL* model. The parameters are in the same order as Fig 5 in the main text.
(TIF)
