# Supplementary material for: Bayesian phylodynamic inference with complex models
Source: PLoS Comput Biol. 2018 Nov 13;14(11):e1006546. doi: 10.1371/journal.pcbi.1006546 (PMC6258546; doi:10.1371/journal.pcbi.1006546)
Supplement: S1 Text — (PDF) [file pcbi.1006546.s001.pdf]

Bayesian phylodynamic inference with complex models

Erik M. Volz[1*], Igor Siveroni[1],

1 Department of Infectious Disease Epidemiology and MRC Centre for Outbreak Analysis and Modelling, Imperial College London, United Kingdom

S1 Text: Structured coalescent likelihood and approximations

In this section we review the approximate structured coalescent model described in [1] and describe extensions designed to improve accuracy and reduce computational cost.

Derivation of PL2

The structured coalescent defines a discrete Markovian stochastic tree-building process in retrospective/continuous time [2]. In it's most general form, the process defines the potentially time and state dependent rates that 1) a lineage changes demes and 2) the rate of 'coalescence', i.e. the rate that two or more lineages form a single lineage at a node. In this section, we explain how the (F, G, Y) model-based coalescent is related to the more general process and approximations involved in computing the fast likelihood. We consider a structured coalescent for a bifurcating genealogy that has the following events and rates:

- Deme change. A lineage changes deme from k to l at the rate $M_{kl}(t)$.
- Coalescence. A lineage in deme k 'gives birth' to a lineage in deme l at the rate $\phi_{kl}(t)$ giving rise to an ancestor lineage in deme k.

The vast majority of previous work on these models has been focused on phylogeographic applications and therefore consider only the special case $\phi_{kl} = 0$ if $k \neq l$, i.e. lineages only coalesce if they are co-located in the same deme. In these models, we depart from the usual approach and specify different coalescence rates ϕ_{kl} and ϕ_{lk} which yield different types of ancestor lineages if $k \neq l$. Note that the total rate of coalescence of a lineage i in deme k and a lineage j in deme l would be $\phi_{kl} + \phi_{lk}$. Most previous work has also focused on the special case of constant rates, so that M and ϕ are independent of time. Epidemiological and ecological models will often have both time-dependent rates and non-zero off-diagonal elements in ϕ.

At any time in the course of the tree-building process, a set of 'extant' lineages $\mathcal{A}(t)$ with cardinality $A(t)$ will have configuration $S(t) = (s_1, \cdots, s_{A(t)})$ where $s_i \in 1 : m$ is an integer index of m demes. We will also use the notation $A_k(t)$ to denote the number of extant lineages in deme k. The likelihood of a genealogy under the structured coalescent is defined as that of a point process with competing rates for different types of events (coalescence or deme-change). Note that we will also allow rates of coalescence and deme-change to be time and state-dependent. The probability density of a genealogy produced by this process is easily computed provided that the configuration $S(t)$ is known at all time points [3, 4].

Additional notation is required to specify this density:

- In a rooted bifurcating genealogy with n tips, there are $2n - 2$ lineages. We index these such that $i \in 1 : n$ corresponds to terminal branches of the genealogy, and $i \in (n + 1) : (2n - 2)$ is ancestral to an internal node of the tree.

- $\mathcal{C} = \{(t_c^a, i_c, j_c)\}_{c=1:(n-1)}$ is the set of internal nodes represented by a tuple and each also correspond to coalescent events. The indices i and j correspond to the daughter lineages of the internal node, and t^a is the time when the internal node occurs
- $T_S = (t_1, \cdots, t_n)$ denotes times of sampling. We assume the state at time of sampling $s_i(t_i)$ is known.
- $\mathcal{X} = \{(t_x^s, k_x)\}_{x=1:n}$ denotes the set of sample events represented by a tuple. The time of sampling is t^s and the observed deme at time of sampling is k.
- $\mathcal{D} = \{(t_d, k_d, l_d)\}_{d=1:N_d}$ denotes the set of N_d deme-change events represented by a tuple. The time of the event is denoted t_d. The initial deme is k and the deme following the change is l.
- Let $T = (t_1, \cdots, t_N)$ denotes the ordered sequence of all event times (sampling, migration, or coalescence) in the tree building process.

Note that the total coalescence rate between lineages i and j given known lineage-deme configuration S is

$$\lambda_{ij}(t)|S = \phi_{s_i(t),s_j(t)}(t) + \phi_{s_j(t),s_i(t)}(t)$$

We can define the cumulative hazard of migration or coalescence between consecutive time points in T. Note that the configuration S is fixed during this period, but migration and coalescence rates may vary. The cumulative hazard over an interval is:

$$\begin{aligned}\Omega_a|S = \int_{t=t_a}^{t_{a+1}} & \sum_{i \in \mathcal{A}(t)} \sum_{k}^{m} M_{s_i(t),k} \\ & + \sum_{i \in \mathcal{A}(t)} \sum_{\substack{j \in \mathcal{A}(t) \\ j \neq i}} \phi_{s_i(t),s_j(t)}(t)\ dt \end{aligned} \tag{1}$$

The joint probability density of the genealogy and deme-configuration $S(t)$ can now be defined:

$$\begin{aligned} p(\mathcal{G}, S|M, \Phi) = & \prod_{\alpha=1}^{N-1} e^{-\Omega_i|S} \\ & \times \prod_{\mathcal{C}} \lambda_{ij}(t^a)|S \\ & \times \prod_{\mathcal{D}} M_{kl}(t^d) \\ & \times \prod_{\mathcal{X}} p(t^s, k) \end{aligned} \tag{2}$$

The first line provides the probability of no events occurring in each internode interval, the second and third lines provide the rates of each coalescent and deme-change event, and the last line describes the density of the sample times and sample states [5]; in most applications there is not a model of the sampling process or prior information to establish this density, and it is not included.

Since in practice the configurations $S(t)$ are not observed except at times of sampling, estimation of migration rates and/or effective population sizes then requires a strategy for marginalizing over latent $S(t)$, and this is typically done by MCMC simulation [4]. This entails a large computational cost of integrating over a vary large

configuration space [6], so instead we pursue a strategy of developing equations to marginalize over lineage states and will derive an approximate likelihood in terms of the probability that a lineage is in a particular deme. Our strategy is to derive equations for $p_{ik}(t) = p(s_i(t) = k)$ at all times t over the history of each lineage i. Deriving equations for the joint probability of s_i and s_j is complicated, so we make the simplifying approximation that the conditional probability of state j is equal to the marginal: $p(s_j = l|s_i = k) \approx p(s_j = l)$. We may then compute rates of deme-change for lineage i and the rate of coalescence of i with all other lineages. Where the configuration is unknown, we have

$$\begin{aligned}
\lambda_{ij}(t) &= \sum_k^m \sum_l^m \phi_{kl}(t) \left(p(s_i(t) = k) p(s_j(t) = l | s_i(t) = k) + p(s_i(t) = l) p(s_j(t) = k | s_i(t) = l) \right) \\
&\approx \sum_k^m \sum_l^m \phi_{kl}(t) \left(p(s_i(t) = k) p(s_j(t) = l) + p(s_i(t) = l) p(s_j(t) = k) \right) \qquad (3) \\
&= p_i' \Phi p_j + p_j' \Phi p_i
\end{aligned}$$

We compute the cumulative hazard of coalescence in internode intervals as before, but considering only times of sampling and times of coalescence. In contrast to the above equation for $\Omega_i|S$, the lineage configuration will change over the course of each internode interval. The symbol $\mathcal{S}(t)$ denotes the set of all possible lineage-deme configurations at time t and $p(S(t))$ is the marginal probability of a particular configuration S.

$$\Omega_i = \int_{t_i}^{t_{i+1}} \sum_{S \in \mathcal{S}(t)} p(S(t)) \sum_{i \in \mathcal{A}(t)} \sum_{\substack{j \in \mathcal{A}(t) \\ j \neq i}} \left(\phi_{s_i, s_j} + \phi_{s_j, s_i} \right) / 2 \; dt \qquad (4)$$

We introduce the indicator function $I(x = y)$ which evaluates as one if and only if x equals y and is zero otherwise. Rearranging the order of summation

$$\Omega_i = \int_{t_i}^{t_{i+1}} \sum_{i \in \mathcal{A}(t)} \sum_{\substack{j \in \mathcal{A}(t) \\ j \neq i}} \sum_k^m \sum_l^m \sum_{S \in \mathcal{S}(t)} p(S(t)) \left(\phi_{kl} + \phi_{lk} \right) I(s_i = k) I(s_j = l)/2 \; dt \qquad (5)$$

Recognizing that $p(s_i = k)p(s_j = l|s_i = k) = \sum_{S \in \mathcal{S}(t)} p(S(t)) I(s_i = k) I(s_j = l)$, we can simplify the preceding:

$$\begin{aligned}
\Omega_i &\approx \int_{t=t_i}^{t_{i+1}} \sum_{i \in \mathcal{A}(t)} \sum_{\substack{j \in \mathcal{A}(t) \\ j \neq i}} \frac{p_i'(t)\Phi(t)p_j(t) + p_j'(t)\Phi(t)p_i(t)}{2} \; dt \\
&\approx \int_{t=t_i}^{t_{i+1}} \sum_{i \in \mathcal{A}(t)} \sum_{\substack{j \in \mathcal{A}(t) \\ j \neq i}} \lambda_{ij}(t)/2 \; dt \qquad (6)
\end{aligned}$$

Thus we can avoid computing the full intractable density $p(S(t))$ and use the approximation for $\lambda_{ij}(t)$ in equation 3 provided that we can solve for the marginal probabilities $p(s_i = k)$ and it is shown how to do that below.

Marginalising over all possible configurations in $\mathcal{S}(t)$, the density of a genealogy is

$$p(\mathcal{G}|M,\phi) = \prod_{\alpha=1}^{N-1} e^{-\Omega_i} \times \prod_{\mathcal{C}} \lambda_{ij}(t^a) \times \prod_{\mathcal{X}} p(t^s, k) \tag{7}$$

We now derive a system of $m+1$ equations for the probability $p_{ik}(t) = p(s_i(t) = k)$. These equations are defined in terms of the (F, G, Y) model decomposition and the reader should review the main text and reference [1] for how this works. As stated here, these equations are self-consistent, and it is only of importance that the rates of different migration and coalescent events through time are pre-specified. The events which can modify the deme of lineage i when tracing its history backwards in time are

- Migration. Lineage i will move from deme k to l at a rate $G_{lk}(t)/Y_k(t)$.
- Birth. A lineage not ancestral to the sample (not represented by a lineage in the tree) may give birth to lineage i. The deme of lineage i would then be the deme of the lineage which gave birth to i.
- Coalescence. We must condition on the event that no coalescence is observed over each internode interval.

We find it necessary to distinguish between birth events which result in coalescence and birth events which will not. Note that this is not usually required in phylogeographic coalescent models because birth events would not result in deme change but only in potential coalescence. Collecting the first two types of events which change the deme of a lineage, we can define the following time dependent migration matrix for lineage i:

$$M_{kl}^{(i)}(t) = \begin{cases} \left(G_{lk}(t) + F_{lk}(t)(1 - \frac{1}{Y_l(t)} \sum_{j\in\mathcal{A}(t)} p(s_j(t) = l | s_i(t) = k))\right)/Y_k(t) & \text{if } k \neq l \\ 0 & \text{if } k = l \end{cases}$$

This provides the rate that lineage i migrates from deme k to l at time t. As above, the joint density for s_i and s_j is not tractable, so we use the approximation

$$M_{kl}^{(i)}(t) \approx \begin{cases} \left(G_{lk}(t) + F_{lk}(t)(1 - \frac{1}{Y_l(t)} \sum_{j\in\mathcal{A}(t)} p_{jl}(t))\right)/Y_k(t) & \text{if } k \neq l \\ 0 & \text{if } k = l \end{cases} \tag{8}$$

To account for the effect of coalescence on evolution of the state vector $p_i(t)$, we instead develop equations for the augmented state vector $\tilde{p}_i(t)$, which has $m+1$ elements and where $\tilde{p}_{i,m+1}(t)$ represents the probability that lineage i has coalesced at some time $t' < t$. If t_i represents the initial time of lineage i (the most recent time that i exists on forward time axis), we have

$$\tilde{p}_{ik}(t) = p_{ik}(t_i) \quad k < m+1$$

and $\tilde{p}_{i,m+1}(t_i) = 0$. This vector provides the marginal probability of a 'leaky' continuous time Markov process with $m+1$ states, where $m+1$ is an absorbing state. To retrieve the state vector conditioning on no coalescent event having occurred, we use the identity

$$p_{ik}(t) = \tilde{p}_{ik}(t)/(1 - \tilde{p}_{i,m+1}(t)) \qquad \text{if } k < m+1 \tag{9}$$

The forward equations for the evolution of $\tilde{p}_i$ are found by tabulating the effects of migration, birth, and coalescent events outlined above:

$$\begin{aligned}
\tilde{p}_{ik}(t+\Delta t)|k<m+1 = \tilde{p}_{ik}(t) & \\
& +(\Delta t)\sum_{l\neq k} M_{lk}^{(i)}\tilde{p}_{il}(t) - M_{kl}^{(i)}\tilde{p}_{ik}(t) \qquad \text{“Migration”} \\
& -(\Delta t)\sum_{\substack{j\in\mathcal{A}(t)\\ j\neq i}}\sum_{l}^{m}\tilde{p}_{ik}(t)p(s_j=l|s_i=k)(t)(\phi_{kl}(t)+\phi_{lk}(t)) \qquad \text{“Coalescent”} \\
& +\mathcal{O}(\Delta t)
\end{aligned} \tag{10}$$

Using the approximation $p(s_j = l|s_i = k) \approx p_{jl}(t)$ and simplifying this becomes

$$\begin{aligned}
\tilde{p}_{ik}(t+\Delta t)|k<m+1 = \tilde{p}_{ik}(t) & \\
& +(\Delta t)\sum_{l\neq k} M_{lk}^{(i)}\tilde{p}_{il}(t) - M_{kl}^{(i)}\tilde{p}_{ik}(t) \qquad \text{“Migration”} \\
& -(\Delta t)\sum_{l}^{m}\tilde{p}_{ik}(t)(\phi_{kl}(t)+\phi_{lk}(t))(A_l(t)-p_{il}(t)) \qquad \text{“Coalescent”} \\
& +\mathcal{O}(\Delta t)
\end{aligned} \tag{11}$$

Note that the state vector p_j is used instead of $\tilde{p}_j$ when computing rates of coalescence since we must condition as well on no coalescent event having occurred on lineage j. Computing the limit $\Delta t \to 0$ and making appropriate substitutions of $M(t)$ and $\phi(t)$ for $F(t)$ and $G(t)$ gives the system of differential equations:

$$\begin{aligned}
\frac{\mathrm{d}}{\mathrm{d}t}\tilde{p}_{ik}(t)|k<m+1 = & \sum_{l\neq k}\tilde{p}_{il}(t)\left(G_{kl}(t)+F_{kl}(t)(1-\frac{1}{Y_k(t)}\sum_{\substack{j\in\mathcal{A}(t)\\ j\neq i}} p_{jk}(t))\right)/Y_l(t) \\
& -\sum_{l\neq k}\tilde{p}_{ik}(t)\left(G_{lk}(t)+F_{lk}(t)(1-\frac{1}{Y_l(t)}\sum_{\substack{j\in\mathcal{A}(t)\\ j\neq i}} p_{jl}(t))\right)/Y_k(t) \\
& -\sum_{l}^{m}\tilde{p}_{ik}(t)\frac{1}{Y_k(t)Y_l(t)}(F_{kl}(t)+F_{lk}(t))(A_l(t)-p_{il}(t))
\end{aligned} \tag{12}$$

The ‘initial’ state of a lineage following coalescence is found according to the model in [1]. If i and j coalesce at a lineage a, $p_{ak}(t)$ is the probability that i was in state k and gave birth to l or vice versa:

$$p_{ak}(t_a) = \sum_{l}^{m}\phi_{kl}\left(p_{ik}(t_a)p_{jl}(t_a)+p_{il}(t_a)p_{jk}(t_a)\right)/\lambda_{ij}(t_a) \tag{13}$$

Derivation of PL1

In [1], the following approximation (denoted “com12”) was presented which required the solution of the following differential equations:

$$\frac{\mathrm{d}}{\mathrm{d}t}p_i^{com12}(t) = R^{com12}(t)'p_i(t) \tag{14}$$

where R^{com12} is the $m \times m$ matrix with elements

$$R_{kl}^{(com12)}(t) = \begin{cases} (F_{lk}(t)\frac{Y_l(t)-A_l(t)}{Y_l(t)} + G_{lk}(t))/Y_k(t) & \text{if } k \neq l \\ -\sum_{z \neq k} R_{kz}^{com12} & \text{if } k = l \end{cases} \tag{15}$$

Note the inclusion of the term $\frac{Y_l(t)-A_l(t)}{Y_l(t)}$ in equation 15, which was an approximation intended to account for the fact that only births from lineages not ancestral to the sample could cause a lineage to change state without resulting in coalescence. The form of this equation was found to provide an accurate approximation to the lineages through time in [1], when solving the system of equations

$$\frac{\mathrm{d}}{\mathrm{d}t}A(t) = R^{com12}(t)'A(t) \tag{16}$$

A drawback of the *com12* model is that it does not condition on all possible events that lineage i may experience during its evolution; namely it does not condition on the observation that no coalescent events occurred during an internode interval. Consequently, this model tends to overestimate the probability that a lineage occupies a deme with a high coalescent rate. This issue was thoroughly explored in a recent investigation by Muller et al. [7] in the special case of phylogeographic models (diagonal $F(t)$, constant rates, constant population size). A preliminary model which also addresses this issue in was introduced by Volz in the *rcolgem* R package [8] which was also described in [9], and we build on the approach developed therein which explicitly computes the cumulative probability that lineage i has coalesced.

We define the more simple rate matrix $R^{PL1}(t) = (F(t) + G(t))/Y(t)$.

$$R_{kl}^{(PL1)}(t) = \begin{cases} (F_{lk}(t) + G_{lk}(t))/Y_k(t) & \text{if } k \neq l \\ -\sum_{z \neq k} R_{kz}^{(PL1)} & \text{if } k = l \end{cases} \tag{17}$$

Now we collect state vectors into a matrix $\tilde{P}^{(PL1)}(t)$ with m rows and columns $(\tilde{p}_1, \cdots, \tilde{p}_{2n-2})$. And we will define a matrix Ψ with the same dimensions as $\tilde{P}^{PL1}$ which gives the rate of coalescence in each lineage due to births by that lineage and conditional on membership in each deme. Ψ has elements

$$\Psi_{ki}(t) = \tilde{p}ki(t) \sum_{\substack{j \in \mathcal{A}(t) \\ j \neq i}} \sum_{l}^{m} p_{jl}(t)\phi_{kl}(t) \tag{18}$$

We can now solve for the state vectors with the following system of equations

$$\frac{\mathrm{d}}{\mathrm{d}t}\tilde{P}^{(PL1)}(t) = R'(t)\tilde{P}^{(PL1)}(t) - \Psi(t) \tag{19}$$

Normalizing vectors and computing likelihoods then proceeds as above.

Note that PL1 is a little faster than PL2, but is making an additional approximation: It does not account for the number or type of ancestral lineages when computing the rate that a lineage changes state do to birth events (note the way that $F(t)$ enters into the equation for $R(t)$).

Derivation of QL

Unfortunately, the system of equations 12 and 19 can be slow to solve since it requires recursion over extant lineages (twice) and $m + 1$ ancestral states. We therefore provide an additional approximation which greatly reduces computational cost. As above, $\tilde{p}_i$

represents an augmented state vector with $m + 1$ elements where $\tilde{p}_{i,m+1}$ represents the probability that lineage i has coalesced. Note that $m + 1$ is an absorbing state which increases at the rate $\lambda_{i\cdot}(t)$. We define $Q(t, T)$ to be the $m \times m$ matrix of transition probabilities such that

$$\tilde{p}_i^{QL}(\tau) = Q'(t, \tau)\tilde{p}_i^{QL}(t)$$

and $\tilde{p}_i^{QL}$ provides the length m unnormalized state vector excluding the $m + 1$'th element. This vector can be renormalized giving the unit vector $p_{ik}^{QL} = \tilde{p}_{ik}^{QL}/|\tilde{p}_i^{QL}|$. We can also approximate the number of lineages in each deme over the interval using

$$A(\tau) = Q'(t, \tau)A(t)/|A(t)| \tag{20}$$

where $t < \tau < T$. Finally, we can modify equation 12 to use the vector $A(\tau)$, avoiding the need to sum over extant lineages. The following matrix provides transition rates between states.

$$R_{kl}^{(QL)}(t) = \begin{cases} (F_{lk}(t) + G_{lk}(t))/Y_k(t) & \text{if } k \neq l \\ -Q'_{1:m,k}\Phi A(t) - A(t)'\Phi Q_{1:m,k} - \sum_{z \neq k} R_{kz}^{(QL)} & \text{if } k = l \end{cases} \tag{21}$$

Note that the diagonal of the rate matrix also includes the approximate rate that a lineage will coalesce given that it begins the internode interval in each of the m demes. Thus this is a 'leaky' Markov process and the probabilities in each column of Q will not in general sum to one.

Initially $Q(t, t) = I$ is the identity matrix. The matrix $Q(t, T)$ is computed by solving the equations

$$\frac{\mathrm{d}}{\mathrm{d}\tau}Q(t, \tau) = R^{QL}(\tau)'Q(t, \tau) \tag{22}$$

Therefore only m^2 differential equations need to be solved over every internode interval. Note that the rate of coalescence in equation 21 exceeds that in 12, so that this fast approximation will slightly over-estimate the probability that a lineage coalesces over an interval.

References

1. Volz EM. Complex population dynamics and the coalescent under neutrality. Genetics. 2012;190(1):187–201.

2. Wakeley J. Coalescent theory: an introduction. 575: 519.2 WAK; 2009.

3. Beerli P, Felsenstein J. Maximum-likelihood estimation of migration rates and effective population numbers in two populations using a coalescent approach. Genetics. 1999;152(2):763–773.

4. Vaughan TG, Leventhal GE, Rasmussen DA, Drummond AJ, Welch D, Stadler T. Directly Estimating Epidemic Curves From Genomic Data; 2017.

5. Volz EM, Frost SD. Sampling through time and phylodynamic inference with coalescent and birth–death models. Journal of The Royal Society Interface. 2014;11(101):20140945.

6. De Maio N, Wu CH, O'Reilly KM, Wilson D. New Routes to Phylogeography: A Bayesian Structured Coalescent Approximation. PLoS Genet. 2015;11(8):e1005421.

7. Müller NF, Rasmussen DA, Stadler T. The Structured Coalescent and Its Approximations. Molecular biology and evolution. 2017;34(11):2970–2981.

8. Volz EM. rcolgem: statistical inference and modeling of genealogies generated by epidemic and ecological processes. R package version 0.0. 5/r154. 2016;.

9. Volz EM, Ndembi N, Nowak R, Kijak GH, Idoko J, Dakum P, et al. Phylodynamic analysis to inform prevention efforts in mixed HIV epidemics. Virus Evol. 2017;3(2):vex014.
