# Supplementary figures and images for: Bayesian phylodynamic inference with complex models
Source: PLoS Comput Biol. 2018 Nov 13;14(11):e1006546. doi: 10.1371/journal.pcbi.1006546 (PMC6258546; doi:10.1371/journal.pcbi.1006546)

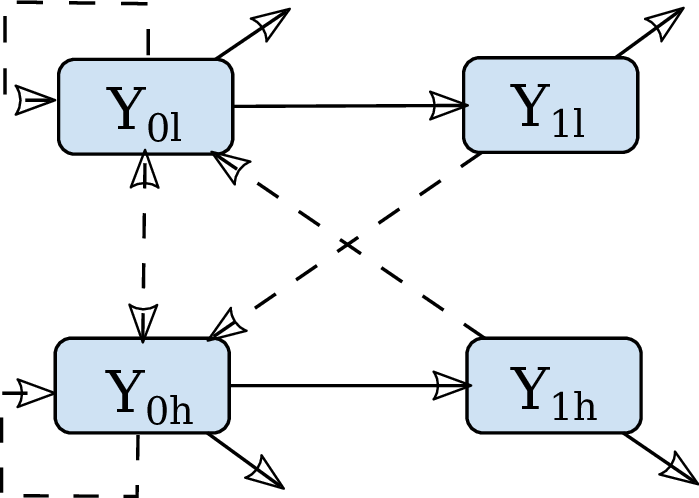

Supplement: S1 Fig — This model has two levels of transmission rate (l and h) and two stages of infection with higher transmission in the first stage. Solid lines represents death or stage progression. Dash lines represent transmissions. (TIF) [file pcbi.1006546.s002.tif]

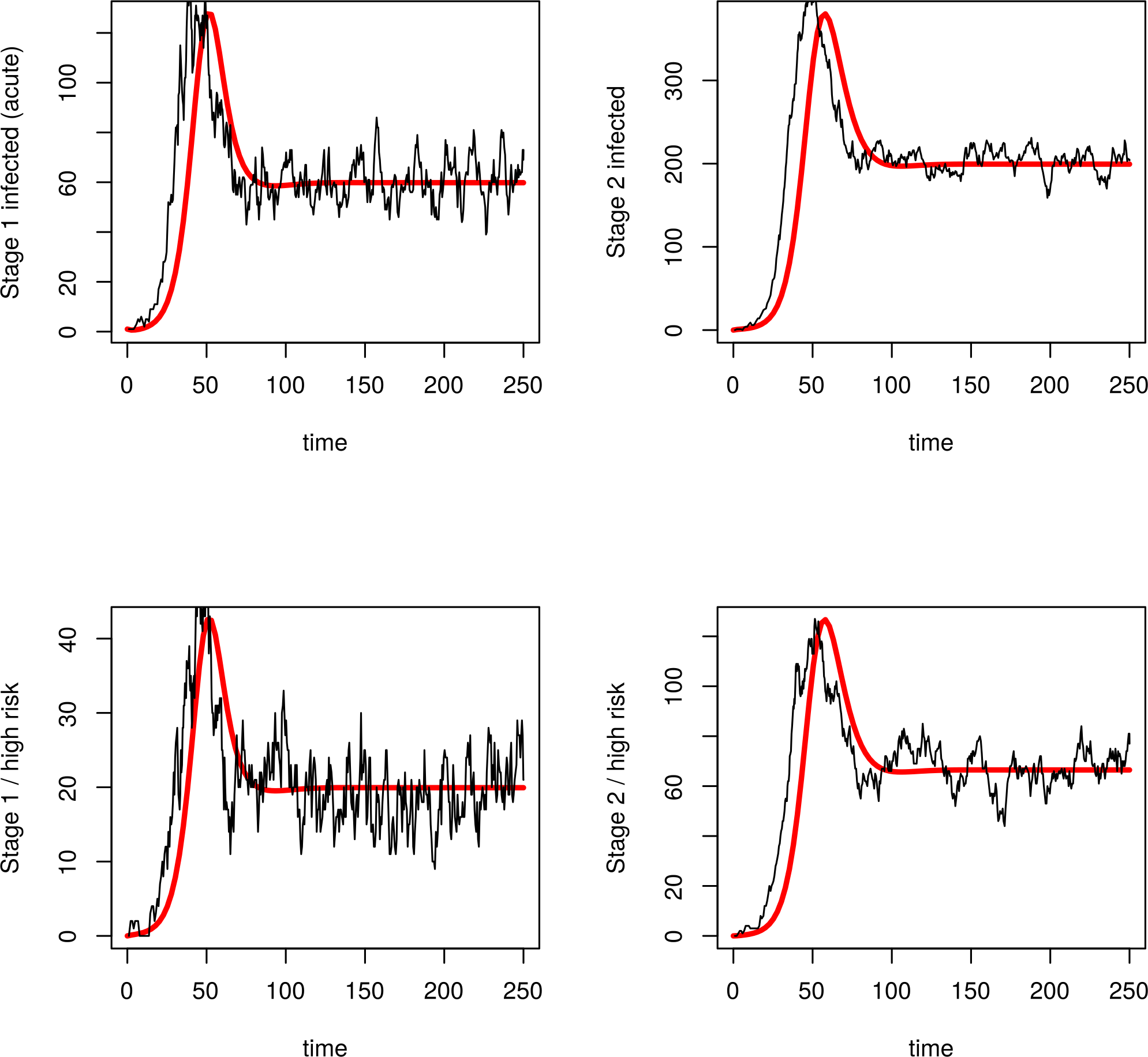

Supplement: S2 Fig — The stochastic epidemic simulation is shown in black and the deterministic ODE model is shown in red. (TIF) [file pcbi.1006546.s003.tif]

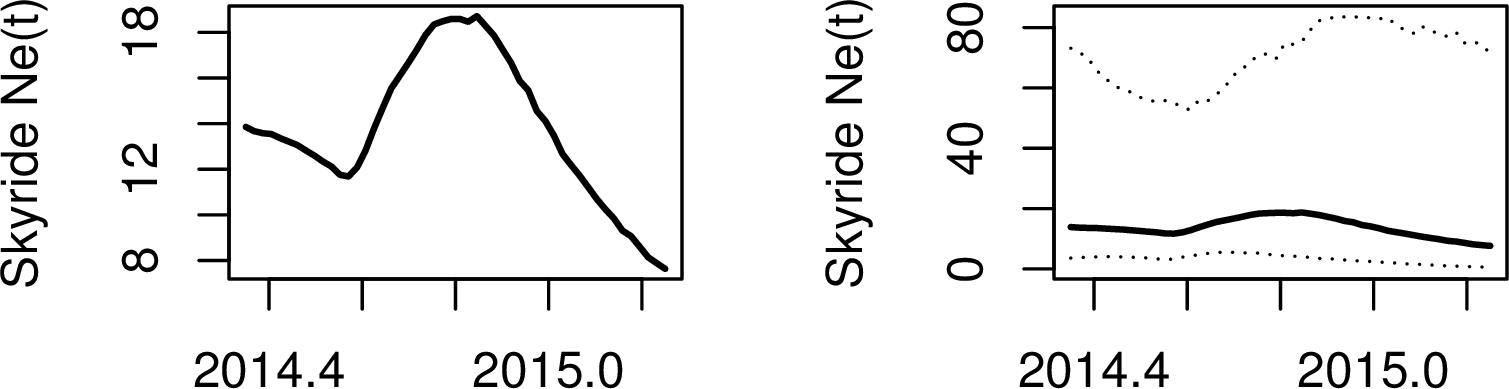

Supplement: S3 Fig — The median posterior estimate is shown in the panel on the left, and the panel on the right shows both the median and 95% credible intervals. (TIF) [file pcbi.1006546.s004.tif]

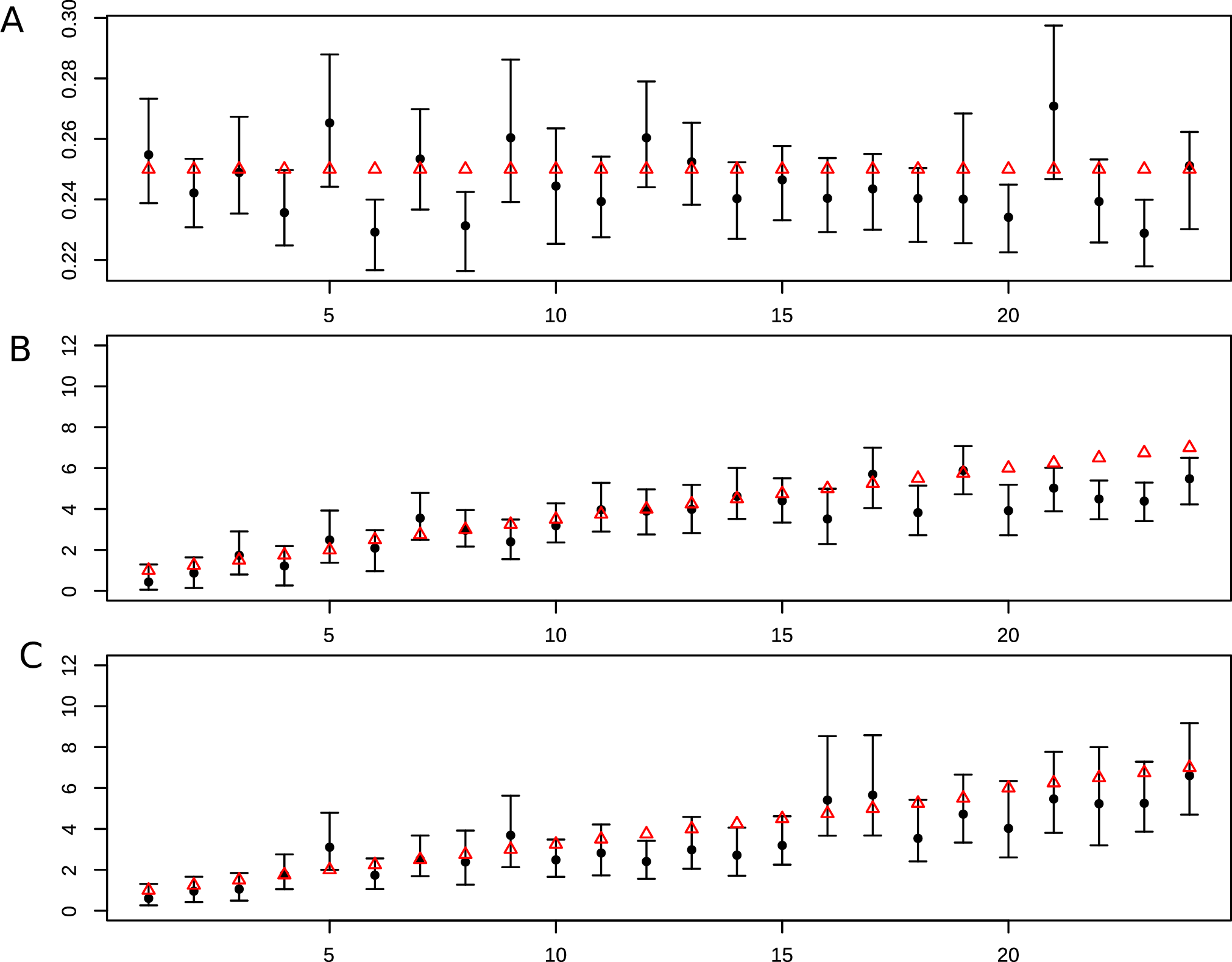

Supplement: S4 Fig — The red points show true parameter value. Top: Transmission rate. Middle: Acute stage transmission risk ratio. Bottom: High risk group transmission risk ratio. (TIF) [file pcbi.1006546.s005.tif]

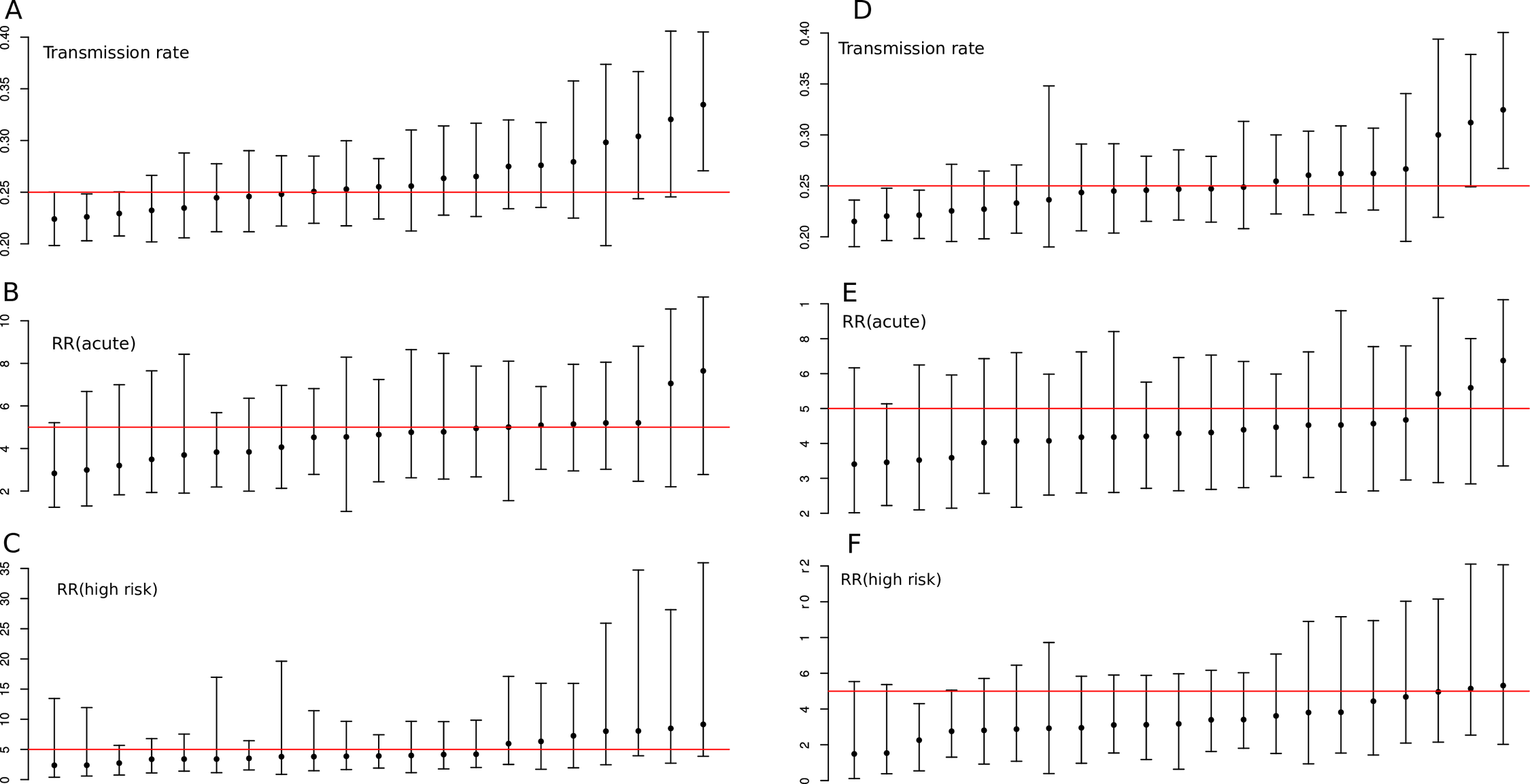

Supplement: S5 Fig — The red line shows the true value. A-C: Results generated using the PL1 model. D-F: Results generated using the QL model. The parameters are in the same order as Fig 5 in the main text. (TIF) [file pcbi.1006546.s006.tif]
